# Wound of the Chest and Liver

**Published:** 1831

**Authors:** 


### Wound of the Chest and Liver.

A Hussar, aged 24 years, was received into the Val de Grace Hospital on the 16th February, in consequence of a sabre-wound, which penetrated between the third and fourth false ribs, on the right side, within two inches of the spine. The sabre, which was large, pierced the diaphragm, and penetrated into the centre of the liver. M. Fournal was immediately summoned, and found the soldier pale, faint, and sick, with feeble pulse and profuse hæmorrhage. The blood which issued from the wound was of a very black colour; the respiration was short and laborious —the extremities cold. Warmth was applied to the feet, and warm acidulated drinks were administered through the night. 17th, in the morning. The pulse was full and quick—the breathing very oppressed. A large venesection—two lavements—total abstinence from food. The venesection was repeated at 3, p. m. He had no sleep the succeeding night; yet the symptoms were somewhat mitigated. 18th, morning. The countenance was yellow —great pain in the region of the wound, with some œdema. Forty leeches were applied to the right side, and the part covered with poultices. The night was passed tranquilly, but without sleep. 19th. Pulse full, soft, regular—pain in the right shoulder and side—the skin deeply tinged yellow—bowels constipated—urine scanty and very red—

wound swelled, but no suppuration apparent. Lavements were exhibited with little effect. Twenty-five leeches around the wound—syncope was the result. The night was good, and some little sleep was obtained. 20th. The state of the patient was still better. There was, as yet, no suppuration about the wound. The yellowness of skin less intense. 21st. The patient is greatly improved in all respects—he has had a good night—and suppuration is established in the wound. Twenty-five leeches, however, were applied around the wound, and the application was followed by syncope. From this time the patient continued to mend, though the yellowness of the surface remained for some time.

---

